# Supplementary material for: Information transmission in a cell monolayer: A numerical study
Source: PLoS Comput Biol. 2025 Feb 21;21(2):e1012846. doi: 10.1371/journal.pcbi.1012846 (PMC11902151; doi:10.1371/journal.pcbi.1012846)
Supplement: S1 Appendix — : Fig A. Front propagation speed. Fig B. Chaotic front spawning. Fig C. Propensity of disruptive events for inert boundary conditions. Fig D. Front speed reduction due to the vicinity of a previous front. Fig E. Histogram of the temporal distance Δt from the expected arrival time to the nearest arrival time, conditioned on whether a front was initiated in the given slot. Fig F. Dependence of Wfail and Wspawn on model parameters. Fig G. Bitrate dependence on the number of E, I, and R substates. Text A with Fig H. Maximum bitrate for non-equiprobable binary symbols. Text B with Fig I. Alternative decoding: Bitrate estimation based on more than one front arrival time. (PDF) [file pcbi.1012846.s001.pdf]

Appendix: Supporting Information

Information transmission in a cell monolayer: A numerical study

Paweł Nałęcz-Jawecki[1], Przemysław Szyc[2], Frederic Grabowski[1],
Marek Kochańczyk[1] and Tomasz Lipniacki[1]*

[1] Institute of Fundamental Technological Research, Polish Academy of Sciences, Warsaw, Poland.

[2] Faculty of Physics, University of Warsaw, Warsaw, Poland.

This file contains the following material:

- **Fig A. Front propagation speed.**
- **Fig B. Chaotic front spawning.**
- **Fig C. Propensity of disruptive events for inert boundary conditions.**
- **Fig D. Front speed reduction due to the vicinity of a previous front.**
- **Fig E. Histogram of the temporal distance Δ*t* from the expected arrival time to the nearest arrival time, conditioned on whether a front was initiated in the given slot.**
- **Fig F. Dependence of W_{fail} and W_{spawn} on model parameters.**
- **Fig G. Bitrate dependence on the number of E, I, and R substates.**
- **Text A with Fig H. Maximum bitrate for non-equiprobable binary symbols.**
- **Text B with Fig I. Alternative decoding: Bitrate estimation based on more than one front arrival time.**

Software used for simulations is available on GitHub https://github.com/kochanczyk/qeirq. Scripts used to create the figures together with generated data are available in a separate repository: https://github.com/pSzyc/visavis-seir.

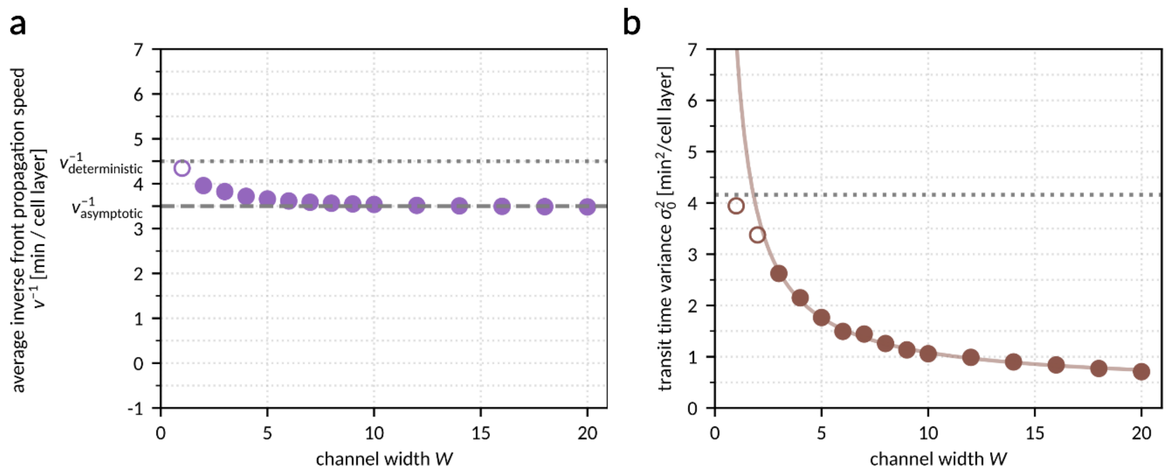

Fig A. Front propagation speed.

a Average inverse front speed as a function of channel width. Dotted line denotes the analytically computed velocity $v^{-1}_{\text{deterministic}}$ = 4.5 min/cell layer, equal to $\langle v^{-1}\rangle$ for $W = 1$. Dashed line denotes the asymptotic inverse velocity of 3.5 min/cell layer.

b Traveling time variance normalized with the channel length, as a function of channel width. Dotted line denotes the analytically computed value $\sigma^2_{0,\text{analytical}} \approx 4.16$ min^2/cell layer for $W = 1$ in the stochastic model. Solid line is the function $\sigma^2_0 = a / W + b$ fitted to data for $W > 2$; best fit coefficients: $a \approx 6.8$ min^2/layer^2, $b \approx 0.4$ min^2/layer.

Data from 30,000 simulations in a channel of length $L = 300$ (filled circles) or $L = 30$ (empty circles).

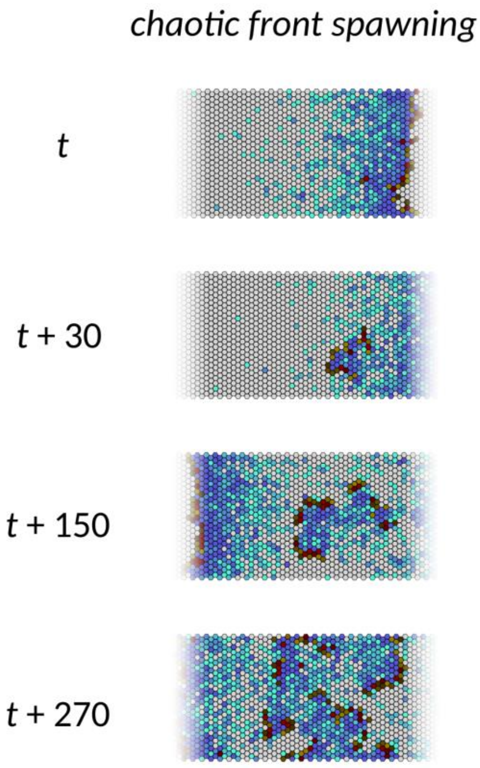

Fig B. Chaotic front spawning. Snapshots from simulations with fronts propagating in directions not parallel to the channel axis. See also S4 Video. Channel width $W = 24$.

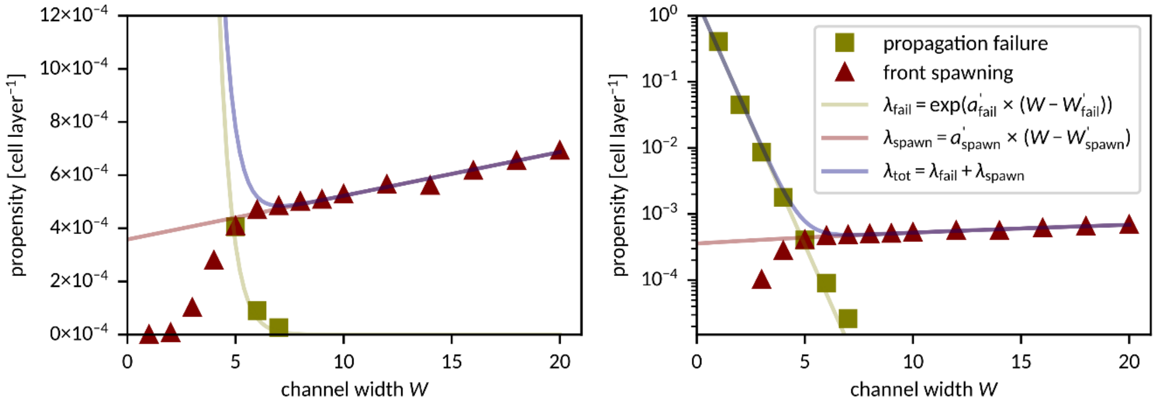

Fig C. Propensity of disruptive events for inert boundary conditions. Inert (non-periodic) boundary conditions along the longer edge are applied. The analysis of disruptive events associated with front propagation corresponds to that in main text Fig 2C.

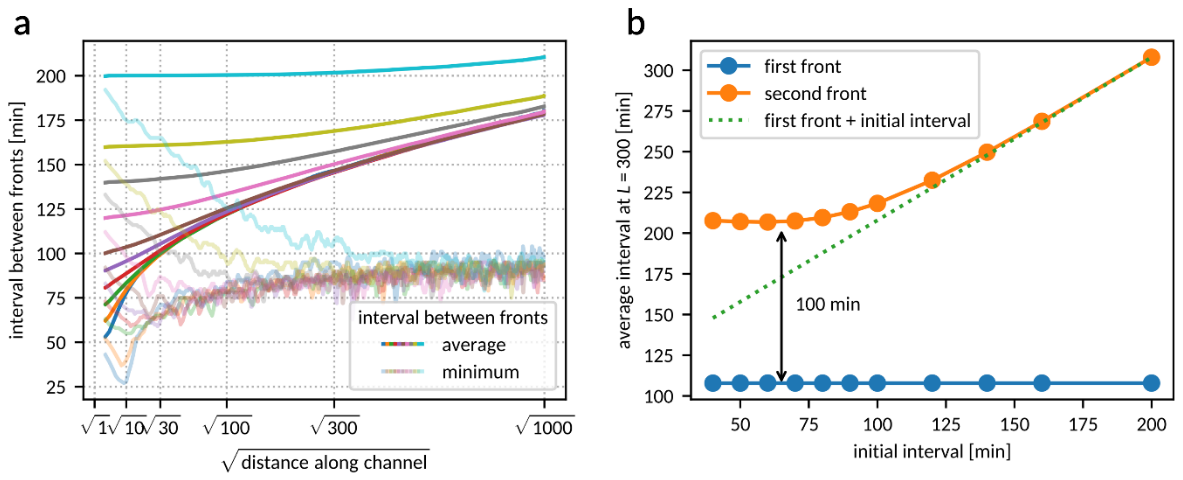

Fig D. Front speed reduction due to the vicinity of a previous front.

a The average (intense-colored curves) and minimal (pale-colored curves) time difference between the moments when two fronts reach a particular distance along the channel. Curves correspond to different initial intervals between the two fronts, ranging from 50 min to 200 min. N = 30,000 simulations per curve.

b Time when each of the two fronts reaches the end of the channel (L = 30) as a function of the initial interval.

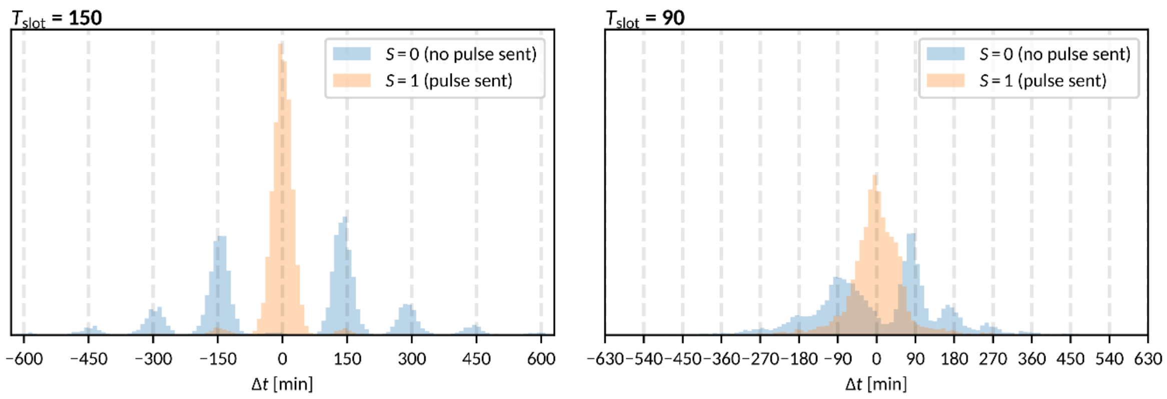

Fig E. Histogram of the temporal distance Δ*t* from the expected arrival time to the nearest arrival time, conditioned on whether a front was initiated in the given slot. Conditional entropy H(S | Δt) was used to estimate the amount of information lost during front propagation through the channel. Simulations as in Fig 4. Channel width $W = 6$, channel length $L = 300$ and two different inter-slot intervals were used. Kinetic parameters were set to the nominal values (Fig 1B).

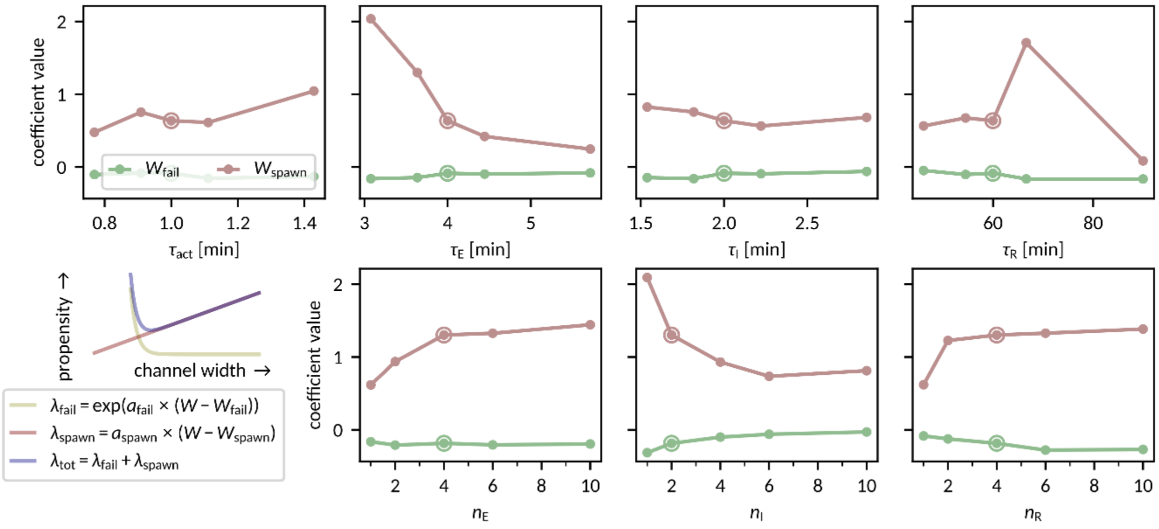

Fig F. Dependence of W_{fail} and W_{spawn} on model parameters. The encircled dots in each panel correspond to the nominal parameter set given in Fig 1B.

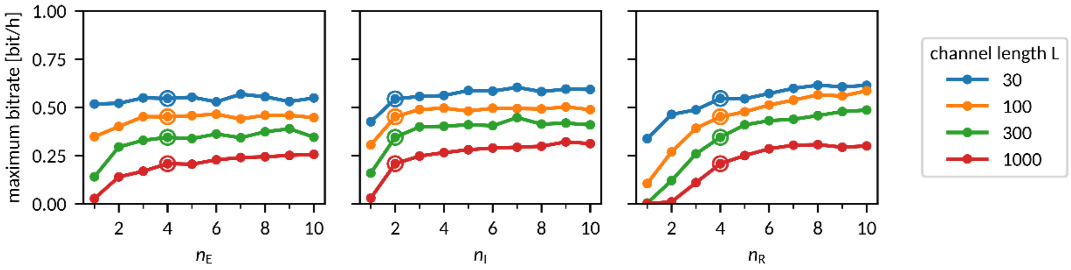

Fig G. Bitrate dependence on the number of E, I, and R substates. The encircled dots in each panel correspond to the nominal parameter set given in Fig 1B.

Text A

Maximum bitrate for non-equiprobable binary symbols.

Throughout the paper, we restricted ourselves to binary protocols with equiprobable ($q = ½$) binary symbols $S \in \{0, 1\}$. Such protocols maximize the information transmission rate in the case when the confusion matrix is symmetric. In our case, the symmetricity of the confusion matrix is broken by the possibility of front extinction, due to which '0's are transmitted more reliably than '1's. In such a case the information transmission rate is maximized for $q < ½$, however the difference with respect to $q = ½$ is marginal, Fig H panel a.

In the case of short channels ($L = 30$), standard deviation of transit time $\sigma_{\mathrm{transit}}$ is small, and sent information is mostly lost due to front propagation failure. We may see that for $L = 30$ the substantially higher information transmission rate may be attained for $q \approx 0.2$ and $T_{\mathrm{slot}} \approx 50$ min much shorter than refractory time $T_{\mathrm{R}} \approx 96.6$ min, Fig H panel b. In this case, two following '1's cannot be transmitted, and the small value of q is beneficial as it implies low probability of sending two '1's in a row. Based on our previous study [1] we know that a protocol in which $\sigma_{\mathrm{transit}} < T_{\mathrm{slot}} < T_{\mathrm{R}}$ but sending two '1's in a row is forbidden, results in even (somewhat) higher information transmission rate.

For longer channels the information transmission rate obtained for $q < ½$ is only marginally higher than that obtained for $q = ½$.

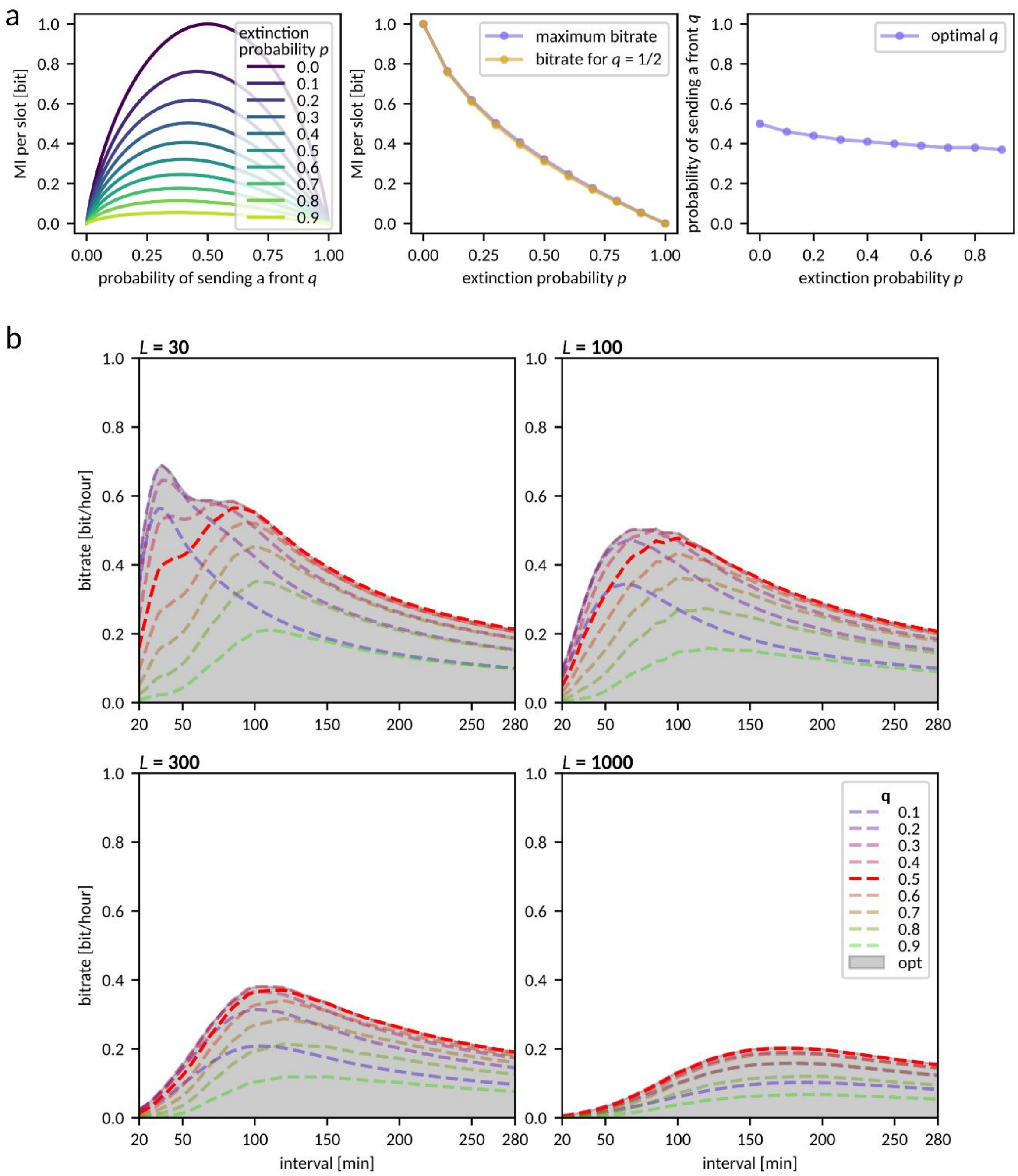

Fig H. Maximum bitrate for non-equiprobable binary symbols.

a Theoretical prediction of the bitrate in a binary protocol with fronts sent with probability q, assuming various probabilities of front extinction p.

b Measured bitrate for different front sending probabilities q as a function of T_{slot}. Shadowed area depicts the bitrate for optimized q.

Text B

Alternative decoding: Bitrate estimation based on more than one front arrival time.

In the main text, the bitrate was estimated based on the arrival times of individual fronts. One could argue, however, that inference based on several consecutive fronts may yield higher bitrate estimates. Using more fronts could help discern whether a given received front originated from a true sent front or rather was spawned in a disruptive event. To verify the extent to which the information transmission rate can be improved in this way, we recomputed the bitrate estimates based on the arrival time of the front closest to t_{expected} and the arrival time of the preceding front (see Fig I below). While the bitrate increase is marginal for long inter-slot intervals, for intervals below T_{R} the information transmission rate estimate is noticeably higher, especially in short channels. For L = 30, where $\sigma_{\text{transit}} \approx 7$ min is much smaller than $T_{\text{R}} \approx 100$ min, a secondary local bitrate maximum appears close to $T_{\text{R}}/2$. In this regime, if two fronts are sent in subsequent slots, the latter will almost certainly be lost. However, this regime allows for time intervals of 3/2 T_{R}, 5/2 T_{R}, 7/2 T_{R}…, which increases the input information rate. Decoding based on two arrival times helps to distinguish whether a front was not received when expected due to it not being sent or because the channel was blocked by a front sent in the previous slot. Taking more than two front arrivals into account does not bring any further benefit (see Fig I below).

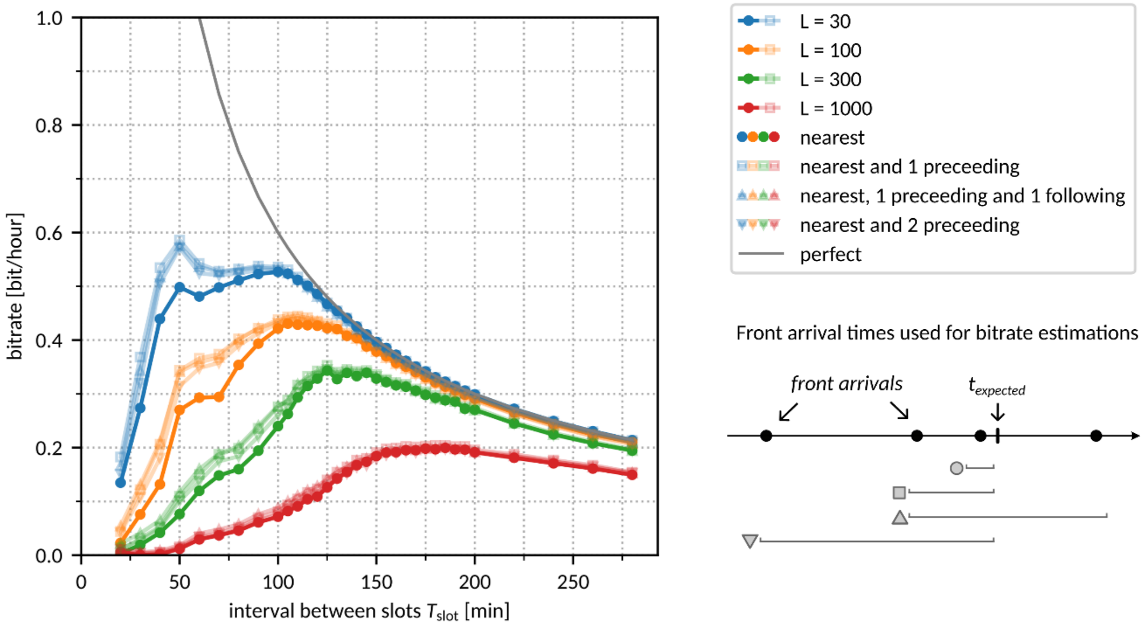

Fig I. Bitrate estimation based on more than one front arrival time. Figure corresponding to Fig 4A in the main text.

References

1. Nałęcz-Jawecki P, Gagliardi PA, Kochańczyk M, Dessauges C, Pertz O, Lipniacki T. The MAPK/ERK channel capacity exceeds 6 bit/hour. PLOS Computational Biology. 2023;19: e1011155. doi:10.1371/journal.pcbi.1011155
