# Supplementary material for: Information transmission in a cell monolayer: A numerical study
Source: PLoS Comput Biol. 2025 Feb 21;21(2):e1012846. doi: 10.1371/journal.pcbi.1012846 (PMC11902151; doi:10.1371/journal.pcbi.1012846)
Supplement: Attachment — Submitted filename: PCOMPBIOL-D-24-01158_report.pdf [file pcbi.1012846.s006.pdf]

Report on “Information transmission in a cell monolayer: A numerical study” by Lipniacki and colleagues

for PLoS Computational Biology

September 16, 2024

1 Synopsis

The authors study a cellular automaton model representing the propagation of spatiotemporal waves of MAPK/ERK activity in a monolayer of interacting cells. The model resembles a stochastic SIR (susceptible, infected, recovered) epidemic model (with loss of immunity) on a lattice [1], or the classic Bak-Chen-Tang (BCT) forest fire model [2], albeit with additional substates. The authors numerically study the propagation, branching, and failure of wavefronts seeded at one end of a long, narrow two-dimensional domain, with a particular focus on the effects of changing the domain width. In order to study how well signals propagate in the simulated system, the authors experiment with train of pulses initiated either by a rhythmic pattern of excitation at one end of the domain, or else by a random binary sequence of excitations with different time intervals. In the latter case they estimate an information transmission rate, roughly the mutual information between pulse initiation at one end of the domain and pulse arrival at the other end of the domain.

On the one hand, the system studied by the authors is interesting as a stochastic cellular automaton system, and the authors conduct a very thorough study, although some of the details of their analysis could be presented more clearly. On the other hand, it is not very clear why the noisy propagation of a traveling wave through a long thin strip of tissue is relevant to understanding the functioning of a MAPK/ERK signaling biologically; moreover, the rationale for using an information theoretic framework does not seem very well motivated.

Since my expertise is more in modeling and applications of information theory in neural systems than in MAPK/ERK systems, I will mainly focus this report on the information theoretic aspects of the system.

2 Major issues

1. It is not clear why estimating information rates is relevant to the problem of understanding MAPK/ERK signaling in the kind of system the authors consider. According to the paper,

MAPK/ERK activity is important for "long-range communication" in tissues regenerating after, say, injury. (The authors estimate a maximal bitrate for their model system at around 0.5 bit/hour, cf. Fig. 4E. Is there any biological significance to this number?) The presence of spatiotemporal traveling waves does not by itself imply that there is a "communications system" at work.[1] Is there really a "receiver" in the MAPK/ERK system that is waiting to receive a signal transmitted through the tissue from a spatially distant "sender" selecting a message from a stochastic ensemble of possible messages? Or is the MAPK/ERK system merely another of many examples of a spatially extended excitable system that can support traveling waves? In order to justify the use of information rates as a quantitative metric of the behavior of the MAPK/ERK system, the authors should better motivate why the information theoretic framework is relevant to this system.[2] If the important function is *propagation* of MAPK/ERK activation more than *communication* of a "message," then another framework such as signal-detection theory [6] or ideal-observer analysis [7] might be more relevant.

2. The calculation of "information rates" for long thin strips of tissue seems like a dubious form of quantification because (if I understand correctly) in real systems (which are not thin strips but more likely to be tissue regions that are both broad and long?) the propagating wave fronts break up into chaotic spiral front patterns that are not "carrying information" from a sender to a receiver. Do the authors have specific systems in mind where the "tissue channel" connecting the tissue where activation waves are initiated and propagate linearly to another region where they are somehow needed to arrive in a regular temporal sequence? In short, is there a biological system that looks and acts like the authors' model system (a long thin strip)? If not, the biological relevance of the model and its analysis might not be sufficient for this journal.

3. A single-use communications channel is specified by a source random variable X with distribution $P_X(X)$, a receiver random variable Y and a conditional probability distribution $P(Y \mid X)$ that characterizes the channel. For a given choice of the input distribution P_X one obtains the mutual information $I(X : Y) = H(Y) - H(Y \mid X) = H(X) - H(X \mid Y)$. The capacity of the channel is the maximum (or, technically, the supremum) of $I(X : Y)$ over all allowable input distributions P_X. In a repeated-use communications channel, like the system considered by the authors, the input is a stochastic process X_t, one considers the mutual information rate $\lim_{T\to\infty} \frac{1}{T} I(X_0^T : Y_0^T)$ where X_0^T is the trajectory of the process X_t over the time interval $0 \le t \le T$. The capacity is the largest value of the mutual information rate obtained over all allowed input processes (e.g. subject to some constraint). For a system like that considered by the authors one of the difficulties in studying either the information rate or the capacity is *intersymbol interference*, in which transmission of one symbol can be corrupted by the transmission of a later or earlier symbol. In studying the information rate of their channel, the authors restrict the input to a binary sequence of activations with probability $p_{\text{act}} = 1/2$ at fixed time steps. But this is only one of many possible input processes the authors could consider. The problem is that limiting to a single input process, one does not know if the information rate is anywhere near the best you can obtain for the channel.

[1] For example: There are propagating waves of hypoxia and/or ionic imbalance in models of spreading depression and epileptic activity [3, 4]. The brain, as an organ, is involved in "information" and "communication." But I am not aware of anyone trying to estimate the rate at which a propagating spreading depression wavefront conveys "information" from one part of (say) the hippocampus to another. Why not? Because conveying information is not the "purpose" of spreading depression. (According to [3], SD helps prevent seizures, so one might consider that to be its "purpose.")

[2] Cf. Shannon's classic polemic [5].

It is true that the authors vary the time interval T_{slot} between symbol transmissions (the kth symbol being either an activating pulse or the absence of an activating pulse at time $T_k = t_0 + kT_{\text{slot}}$), and estimate the MI rate as a function of T_{slot}. However, I think it would be important for the authors to consider a broader class of inputs. I offer two suggestions, the first perhaps more practical than the second.

(a) Vary p_{act}. Particularly because of intersymbol interference, for a fixed value of T_{slot}, sending activation pulses with probability $p_{\text{act}} < 1/2$ would reduce the entropy rate of the source X_t but it would also reduce the intersymbol interference. These two competing effects could lead to the optimal p_{act} being considerably less than $1/2$.[3]

(b) Since the authors are operating their channel in continuous time, an even more general (but quite natural) class of input processes would be Poisson processes with a specified rate λ_{act}.[4] In this case, instead of making the artificial assumption that the sender and receiver have access to a shared noiseless clock, the timing of activations at the "sending" end of the channel would be a random increasing sequence of times $0 < T_1 < T_2 < \ldots$.

4. Given the restriction to binary input sequences at clocked intervals, the procedure used by the authors to estimate the information transmission rate seems to require further clarification and justification.

(a) The mean front velocity is not well defined (paragraph preceding eq. (7)). I think the authors actually mean the *conditional* mean front velocity, i.e. conditioned on the front arriving at the far end of the channel. (If a front stops propagating in mid-channel, is its velocity zero? Is it included in calculating the mean front velocity?) Similarly, $\langle \tau_{\text{transit}} \rangle$ should be better defined. Since there is a chance of a front never arriving, the expected transit time is probably infinite. That is, for any number $a > 0$, one has the inequality

$$E[\tau_{\text{transit}}] \geq E[\tau_{\text{transit}} \mid \tau_{\text{transit}} < a] P(\tau_{\text{transit}} < a) + a(1 - P(\tau_{\text{transit}} < a)).$$

If there is a finite chance that a front never arrives, then $P(\tau_{\text{transit}} < a) < 1$ for all a. In this case, taking the limit as $a \to \infty$ shows that the expected transit time must be $+\infty$. The remedy is to consider the *conditional* transit time, that is, the mean transit time conditioned on the event that the pulse arrives within a finite time:

$$\langle \tau_{\text{transit}} \rangle \equiv E[\tau_{\text{transit}} \mid \tau_{\text{transit}} < \infty].$$

If the transit time has a density f_{trans} then

$$\langle \tau_{\text{transit}} \rangle = \frac{\int_0^\infty t f_{\text{trans}}(t)\, dt}{\int_0^\infty f_{\text{trans}}(t)\, dt}$$

where the denominator is $P(\tau_{\text{transit}} < \infty) < 1$.

(b) The description of the procedure:

> "...for each ith slot, the difference Δt_i between the closest arrival time (to the expected arrival time of the ith transmitted symbol) and the expected arrival time was measured"

[3] For a simple example of a biologically inspired channel with a similar tradeoff, see [8].

[4] For the entropy rate of the input, i.e. a Poisson process, see [9].

is problematic. Consider the following scenario: slot $k-1$ sends "0"; slot k sends "1"; slot $k+1$ sends "1". Suppose both the kth and $k+1^{\text{st}}$ slots' wavefronts fail to propagate, but a disruptive event near the time of the $k-1^{\text{st}}$ slot leads to an ectopic front arrival at a time somewhere between the expected arrival times for the kth and $k+1^{\text{st}}$ slots. Then according to the procedure, the ectopic arrival would be counted as an arrival originating from *both* the kth and the $k+1^{\text{st}}$ slots. It would contribute twice to the formula (7). Further clarification, or possibly a correction, is required.

(c) After conducting an ensemble of simulations, then for each time slot the authors count the occurrences of certain time differences Δt between each of a list of expected arrival times and the nearest actual front arrival time. If a time difference occurs a certain number of times ($k = 25$ before eq. (7)) or more, then a conditional entropy $H(S_i \mid \Delta t)$ associated with the ith slot is calculated according to one formula (7) but if the time difference occurs fewer than k times, then the authors use a different formula for $H(S_i \mid \Delta t)$. And on the next page we read "In cases where the MI_{slot} estimation was based on the closest front and its predecessor $k = 15$ was used as it provides better performance." The authors do not sufficiently explain the use of two different formulas for the conditional entropy, one above k and another below k. Why not use one of the formulas throughout, for consistency? They do not explain sufficiently why k was chosen to be 25. What happens if k is 10, or 50, or some other arbitrary number? They do not adequately explain why they change k from 25 to 10 in some situations. Too many elements of this procedure seem to be ad hoc.

3 Minor issues

It would have been convenient if the authors had numbered the pages and lines of their manuscript.

1. Author summary: "However, random effects on THE single-cell level..."
2. Author summary: "the refractory time ... must be long with respect TO THE TIME needed for cell activation."
3. Introduction: "This is the case of spatiotemporal" → "This is the case FOR spatiotemporal"
4. Results / Model definition: Please state early in the modeling section whether the implementation of the model is in continuous time or discrete time. Until the mention of Gillespie's algorithm later, in the Methods section, it was ambiguous.
5. Results / Model definition: From the author's presentation it appears they assume that the rate of spontaneous excitation $Q \to E_1$ is identically zero in the absence of stimulation from neighboring inducing cells. Is that realistic? In some communications channels (e.g. Kabanov's Poisson channel [10], which is a more elementary point-process channel, like the one the authors consider) the capacity is infinite unless there is a dark current, i.e. a finite false positive rate, or finite chance of receiving a signal even if one was not sent. Please clarify the assumption that there is not spontaneous excitation.
6. Results / Propagation of a single front / Propagation failure and front spawning: Below eq. (1), "On the contrary" should be "In contrast".

7. Results / Optimal channel width: Eq. (3) is not adequately explained. On the one hand, it seems clear that the probability that a front fails to make it through the channel will depend on the rates of the two failure modes (front stops propagating spontaneously; front is annihilated by a backpropagating front). So the probability of one front in a train failing to reach the other side may be a function of both λ_{fail} and λ_{spawn}. But why should it be simply the sum of these two rates? Is there an unstated assumption that the two failure modes are mutually exclusive? Please justify this expression more carefully.

8. Results / Optimal channel width: Does "uninterrupted front propagation" refer to the propagation of a single isolated front, a single front in an infinite chain, or an entire infinite chain of fronts? Each of these events has different probabilities. In particular, the probability of "uninterrupted front propagation" is strictly zero, if it refers to an infinite series of fronts. At least one of a long series is sure to fail. Please clarify the meaning of the phrase.

9. Results / Propagation of periodically initiated fronts: "fronts trains" should be "front trains" at the end of this paragraph.

10. Results / Information transmission rate / Numerical results: "To assess the bitrate" should be "To estimate the bitrate".

11. Methods / Information transmission rate – numerical estimation: "To measure the information transmission rate" should be "To *estimate* the information transmission rate."

12. Methods / Information transmission rate – semi-analytical predictions: Is eq. (9) the probability of a *single, isolated* front being eliminated? Or the probability of *any* front in a long train being eliminated? Or of a single front in a long train?

13. Methods / Information transmission rate – semi-analytical predictions: In the confusion matrix, eq. (10), setting the top right entry to zero means that there is no possibility of spontaneous activations occurring. This means that the activation rate is strictly zero unless there is a neighboring cell in the inducing state. Is this biologically accurate? (Cf. question 5 above.)

14. Methods / Information transmission rate – semi-analytical predictions: The authors have two expressions for MI_{slot}, both given by eq. (11) but with two different confusion matrices (10) and (17). In order to see the effects of replacing matrix (10) with matrix (17), did the authors compare the two different values of MI_{slot}?

References

[1] David R De Souza and Tânia Tomé. Stochastic lattice gas model describing the dynamics of the sirs epidemic process. *Physica A: Statistical Mechanics and its Applications*, 389(5):1142–1150, 2010.

[2] Per Bak, Kan Chen, and Maya Paczuski. Solitons in the one-dimensional forest fire model. *Physical Review Letters*, 86(11):2475, 2001.

[3] Yina Wei, Ghanim Ullah, and Steven J Schiff. Unification of neuronal spikes, seizures, and spreading depression. *Journal of Neuroscience*, 34(35):11733–11743, 2014.

[4] Bas-Jan Zandt, Bennie ten Haken, Michel JAM van Putten, and Markus A Dahlem. How does spreading depression spread? physiology and modeling. *Reviews in the Neurosciences*, 26(2):183–198, 2015.

[5] Claude E Shannon. The bandwagon. *IRE transactions on Information Theory*, 2(1):3, 1956.

[6] David Marvin Green, John A Swets, et al. *Signal detection theory and psychophysics*, volume 1. Wiley New York, 1966.

[7] Eric E Thomson and William B Kristan. Quantifying stimulus discriminability: a comparison of information theory and ideal observer analysis. *Neural computation*, 17(4):741–778, 2005.

[8] Peter J Thomas and Andrew W Eckford. Capacity of a simple intercellular signal transduction channel. *IEEE Transactions on information Theory*, 62(12):7358–7382, 2016.

[9] JA McFadden. The entropy of a point process. *Journal of the society for industrial and applied mathematics*, 13(4):988–994, 1965.

[10] Yu M Kabanov. The capacity of a channel of the poisson type. *Theory of Probability & Its Applications*, 23(1):143–147, 1978.
