# Supplementary material for: Information transmission in a cell monolayer: A numerical study
Source: PLoS Comput Biol. 2025 Feb 21;21(2):e1012846. doi: 10.1371/journal.pcbi.1012846 (PMC11902151; doi:10.1371/journal.pcbi.1012846)
Supplement: Attachment — Submitted filename: Nalecz-Jawecki et al Response to Reviewers.pdf [file pcbi.1012846.s007.pdf]

Response to Reviewers

We would like to thank all Reviewers for their remarks, as they were useful to improve the manuscript. We added the following new content to the manuscript:

- A new subsection in *Results* entitled *Front propagation in broad channels*, accompanied with Fig 7, in which we show how propagating fronts behave in channels significantly broader than the optimal width and how the information transmission in such channels may be enhanced by relatively modest change of kinetic parameters,
- S1 Text with S8 Fig, in which we investigate whether the information rate can be increased by releasing the constraint that sending 0 and 1 is equiprobable. We found that the information rate can be increased by about 20% in short, but not long channels.
- A new Fig S3, in which we show how the spawning and failure propensities change if periodic boundary conditions are replaced by inert boundary conditions, i.e. when interacting cells are bordered by the biochemically inert cells,
- A new Fig S5, referenced in *Results* subsection *Numerical results* , which illustrates how we perform the bitrate estimation,
- We rearranged Fig 1 to show the distribution of time spent by cells in each functional state (E, I , R) in panel B.

We rewrote the *Information transmission rate - numerical estimation* subsection in *Methods* where it might have been unclear.

Please note that the full code to reproduce the figures is available in the GitHub repository https://github.com/pSzyc/visavis-seir. It was updated to cover the current modifications.

Below, please find our responses to particular concerns of the Reviewers.

Reviewer #1:

> *The authors investigate how information is transmitted through cell monolayers, motivated by earlier experimental observations of the spatiotemporal waves of MAPK/ERK activity in developing and regenerating tissues. Employing a stochastic cellular automaton framework, they evaluate the efficiency of long-range communication by analyzing the rate of information transmission within 2D rectangular cellular channels. The study identifies key factors that disrupt this process, including front propagation failure, the spawning of new fronts, and variability in front velocity. The authors highlight the importance of optimal channel width, which geometrically determines front length, as a critical factor in maximizing information transmission. Moreover, they demonstrate that the timescale of biochemical reactions in the MAPK cascade is critical to maximize information transmission rate in the tissues.*
>
> *The modeling approach is unique for its ability to simplify the complex mechanistic processes underlying ERK activity propagation in tissues, thereby enabling an exploration of how timescales of cellular processes and the geometry of the field influence the success or failure of information transmission across cells. This simplicity, however, is a double-edged sword. While the model may not fully capture the intricacies of biological phenomena, its simplicity allows for a broader examination of the general principles governing the optimal design of biological systems for cell-to-cell information transfer. To fully leverage the strengths of their approach, the authors could have expanded the scope of their analysis and discussion beyond the current ones.*
>
> *I believe this manuscript provides a unique and intriguing contribution that would appeal to the broad readership of this journal. However, as outlined below, there are several critical points where essential information is lacking, and the authors need to address the following points.*
>
> ***Here are some comments for suggested improvements:***
>
> ***1.** The authors have compared the deterministic and stochastic regimes of the QEIRQ model, emphasizing on the stochastic regime. However, the manuscript lacks in detailing the implementation of stochastic fluctuations. In the Model section, it is mentioned that the time scales follow Erlang distributions, yet the specific parameter values are not provided. As Erlang distributions are defined by two parameters and can vary significantly depending on these values, the absence of this information makes it difficult to fairly evaluate the results and conclusions. It is essential for the authors to clarify the model, as without these details, it is impossible to fully understand the nature of the stochasticity they discuss or to accurately assess their findings.*

Response: We assume that each transition between the sub-states (e.g., $E_1 \to E_2$) is Markovian, and thus the transition times are exponentially distributed (with the rate parameters n_E/τ_E, n_I/τ_I, or n_R/τ_R). Consequently, the residence times in the E, I, and R states follow Erlang distributions: Erlang(n_E, n_E/τ_E), Erlang(n_I, n_I/τ_I), and Erlang(n_R, n_R/τ_R) with means τ_E, τ_I, and τ_R, respectively (see new panel B in Fig 1). Note that by increasing n_E, n_I, or n_R we reduce the stochasticity of the system, because the variance of an Erlang(n, n/τ) distribution is inversely proportional to n. In revision, we have included this information in the

main text (lines 112–120) along with plots of the Erlang distributions in the model definition shown in Fig 1.

> ***2.*** *Essential information regarding the numerical simulations, such as boundary and initial conditions, is missing from the Method section. Although the legend of Figure 1B mentions that 'Periodic boundary conditions are applied along the longer edge,' this should be explicitly described in the Method section if it applies to other simulations as well. Detailed information about the simulation settings is necessary to ensure clarity and reproducibility.*

Response: In the first paragraph of Methods (main text lines 628–634) we added the following text: "In all simulations except those for S3 Fig, periodic boundary conditions were applied along the lower and the upper edges. Cells traverse the QEIRQ sequence of (sub)states when activated by a neighboring cell in an I substate. Cells cannot change their locations nor die. State-to-state transitions are shown in Fig 1A and default parameter values are given in Fig 1B."

> ***3.*** *It appears that the authors have applied periodic boundary conditions on the upper and lower sides of the channels in their simulations. While this setting may be appropriate for modeling duct-like structures, it may not be suitable for certain biological tissues. It is crucial to explore how the results might change under different boundary conditions, as system's behavior under the current setting would highly depend on the bond connections between constituent cells or the medium in the domain. Performing this additional numerical investigation would significantly enhance the generality and robustness of the conclusions drawn.*

Response: We used periodic boundary conditions because they allowed us to focus on the effect of the channel width, without effects created by the boundaries. Prompted by your comment, we added a new supplementary figure (see current S3 Fig and main text lines 240–245) that shows how the propensities λ_{fail} and λ_{spawn} depend on the channel width when inert boundary conditions are assumed (i.e., when interacting cells are bordered by biochemically inert cells). You may notice that now both λ_{fail} and λ_{spawn} are larger, and that λ_{spawn} has increased by a substantial additive constant associated with boundary effects. Consequently, the minimum value of the total front elimination propensity, $\lambda_{\mathrm{tot}} = \lambda_{\mathrm{fail}} + \lambda_{\mathrm{spawn}}$, is (about four-fold) higher than for periodic boundary conditions. The modified λ_{fail} and λ_{spawn} can be used to recalculate the information transmission rate (following a derivation in subsubsection *Semi-analytical predictions based on phenomenological analysis*). Since the modified λ_{fail} and λ_{spawn} are substantially higher, the maximum information transmission rate would be substantially lower.

> ***4.*** *The authors claim that the channel width of 6 is optimal; however, this conclusion appears to be highly sensitive to parameter values, particularly those related to stochasticity. To provide a clearer understanding, the authors should first specify the exact parameter sets they used (as also related to my comment #1) and then show how the optimal width varies with changes in the stochastic parameters."*

Response: We show the dependence of the optimal channel width W_{opt} on all kinetic model parameters in Fig 5C. In particular, we demonstrate its dependence on n_E, n_I, and n_R, which are the shape parameters of respective Erlang distributions. As such, these parameters directly control variances (stochasticity) of waiting times of respective transitions.

As already written in response to ***1.***, in the revised first paragraph of Methods (main text lines 628–634) we remind the Reader how the variance of an Erlang distribution depends on its shape parameter, which in the case of our model expresses the number of substates of a compartment. We also added that "[...] the default parameter values are given in Fig 1B" [former Fig 1C].

> ***5.*** *In the last paragraph of subsection Numerical results, the manuscript simply states 'The optimal inter-slot interval changes consistently with the maximum bitrate (Fig 4F)'. The authors should provide a more detailed explanation of the supporting evidence and data related to this point.*

Response: We rephrase this unprecise statement (lines 385–389 in the revised text) to: "The optimal inter-slot interval grows with *W* (Fig 4F), indicating that in broader channels, in which spawning events are more likely, the reliability of information transmission gained by increasing the distance between fronts (which reduces spawning propensity, Fig 3B) is worth the cost of the reduced front frequency".

> ***6.*** *In the final paragraph of the Discussion section, the authors claim that 'Additionally, sensitivity analysis suggests that the MAPK/ERK pathway is "optimized" for efficient information transmission by traveling ERK activity waves.' However, the results presented in this study are based entirely on theoretical models. In addition, the current modeling framework is not fully aligned with the detailed biochemical and mechanical processes elucidated by previous experimental research. Given these limitations, the authors should consider moderating this claim to better reflect the theoretical nature of their findings.*

Response: We moderated this claim, which currently reads: "[the] sensitivity analysis suggests that the time scales associated with signal transmission in the MAPK/ERK pathway are in the range allowing for efficient information transmission by traveling ERK activity waves" (lines 624–626). Now we use the term 'optimality' only in the context of our numerical findings and say that "our analysis indicates that there is an optimal channel width and optimal slot frequency, at which the front propagation is the most efficient and consequently the highest bitrate is achieved" (lines 622-624).

> ***7.*** *In the developing or regenerating fins of zebrafish, as reported by De Simone* et al. *(Nature, 2021, doi: 10.1038/s41586-020-03085-8), concentric waves propagate unidirectionally in 2D space from a specific central point within the fins. Could the authors explain this phenomenon using their current model? How does the model assumption correspond with the ideas proposed in De Simone's paper? Additionally, a theoretical paper by Hayden* et al. *(Biophys J, 2021, doi: 10.1016/j.bpj.2021.05.004) offers relevant insights from a theoretical viewpoint. It would benefit readers to include a discussion on whether and how the current model aligns with the model proposed in Hayden's work.*

Response: We expanded our analysis by studying front propagation in broader channels (see the last subsection of Results). Specifically, we determined the expected number of fronts that can propagate across broad channels before a first spawning event (see new Fig 7). The sensitivity analysis indicates that front spawning propensity grows only linearly with the channel width while it decreases exponentially with the refractory time. Therefore, a relatively modest increase of the refractory time allows for unperturbed transmission of multiple fronts also in broader channels before chaotic front patterns develop. This may explain why the ERK activity waves observed in zebrafish scales, studied by de Simone *et al*. and modeled by Hayden *et al*., are much more stable. This is discussed in the revised main text (lines 615–621).

> ***Minor points:***
> ***8.*** *Figure 5C is not cited in the main text.*

Response: Citation added at the end of “Sensitivity analysis →Disruptive events in single fronts” section (line 459).

> ***9.*** *In the final paragraph of Results, the manuscript states 'the bitrate also grows monotonically with τ_act,' but should this actually be 'the bitrate also decreases monotonically with τ_act'? It looks so in the leftmost panel of Figure 6D. Please verify this.*

Response: Indeed, this was a mistake, which is now corrected (line 502). Thank you for your careful reading.

> ***10.*** *In the second paragraph of Discussion section, the manuscript states ‘capable of transmitting information between membrane (opto)receptors and ERK’. Please explain what the membrane (opto)receptors indicate.*

Response: In the cited paper, information transmission flow is quantified between light-activatable optoFGFR and a translocation-based reporter of ERK activity. We add information about the specific receptor in parentheses (line 553).

Reviewer #2

> *In their manuscript, the authors study how cell–cell communications allow propagation of fronts using a toy model of the ERK pathway. The authors semi-quantitatively describe the failures in front propagation using three modes of failure. Interestingly, the authors find that there is an optimal information transmission rate has a natural optimum. In a periodic setting, when pulses are sent too fast, the information rate can be high, but the refractory period stops front propagation and when the pulses are too slow, the information rate is low but the propagation is faithful.*
>
> *Overall, this is an interesting study. However, the authors describe semi-qualitative*

phenomena without much experimental corroboration (including analysis of past experiments). Given that this manuscript does not identify nondimensional parameters important for the phenomena observed (propagation failure and information transfer) and that it does not explain/fit to previous experimental data, I would like the authors to expand their discussion further to make their manuscript more relevant for the audience. I have 2–3 suggestions that in my opinion will improve the manuscript significantly.

1. *Information transfer calculation: It is perhaps better to have the inter-pulse-interval as a random variable rather than a fixed periodic value. This way, the authors can compute the mutual information rate between the input Markov process and the output Markov process. Perhaps, the authors can discuss this.*

Response: In fact, in the binary encoding, used in our study, the inter-pulse interval (T_{pulse}) is a random variable. What is kept constant (for a given protocol) is the inter-slot interval (T_{slot}). Because pulses are sent independently with probability q (equal ½ for most of the study), the length of the inter-pulse interval is distributed geometrically, with probability $P(T_{\text{pulse}} = nT_{\text{slot}}) = q(1 - q)^{n-1}$. Prompted also by Reviewer #3, we added a supplementary S1 Text in which we consider binary protocols with arbitrary $q \in [0,1]$ to show that for short channels the maximum bitrate is achieved for $q < ½$. In our earlier study (Nałęcz-Jawecki *et al.*, 2023, doi: 10.1371/journal.pcbi.1011155) focused on information transmission in single cells, we also considered protocols with pulse intervals distributed exponentially.

2. *Topology of the network: The topology of the network is quite restrictive. For example, if I understand correctly, once excited, a cell must undergo the full transition to get back to the Q state. That is, the E state is not allowed to go back to the Q state or the Q state is not allowed to skip the E and the I states and directly jump to the R state. The authors should discuss how a less restrictive topology will affect their results.*

Response: The considered topology was chosen to reflect the main MAPK-ERK pathway scheme, in which the signal proceeds through sequential activation of the kinase cascade: the EGFR/SOS/RAS complex, RAF, and MEK (modeled by us as E states) culminates in ERK activation (I states), then transits to the longest refractory phase (R states) in which SOS is inhibited by ERK in order to finally return to the quiescent state (Q) in which the cell can be activated again. For biochemical reasons, neither of the E, I, R, or Q states may be skipped.

In the context of the SEIR epidemic model (in which a Susceptible individual becomes Exposed due to a contact with an Infectious individual and then sequentially becomes Infectious, Resistant, and again Susceptible), the introduction of such shortcuts would be justified. Indeed, it is possible that some E individuals will never become Infectious, either by returning to Susceptible or transiting directly to the Resistant phase. Although not explicitly included in our model, such skipping transitions could be accounted for by increasing the probability of rapid transition through a given state (I, E, or R) state, which can be achieved by reducing the number of intermediate substates. We infer from Fig 5B that such

alterations will strongly disrupt, or even preclude, front propagation, as for a smaller number of intermediated states, the total disruptive event propensity λ_{tot} is much higher.

In the revised manuscript, when introducing the model we wrote (lines 103–104): "Sequential transition through Q, E, I, and R states and appropriate time scales of the residence in the E, I, and R states are crucial to enable wave propagation", but we prefer not to discuss alternative topologies, as it can be misleading for biologically-oriented readers.

> ***3.*** *Heterogeneity: My biggest concern with the work is the lack of heterogeneity. It has been repeatedly shown that cell to cell variability in RTK signaling pathways is dominated by parameter heterogeneity and not by stochasticity (e.g. Gross et al. Cell Systems 2019, Goetz et al. eLife 2024). Yet, the authors assume identical rate parameters for all cells. I suspect that heterogeneity can either hinder or enhance information transfer. I also understand that these calculations are not in the scope of the current work. Therefore, I would like the authors to at least discuss the role of heterogeneity.*

Response: We fully agree that population heterogeneity in many cases is the main source of the observed variability. We added the following paragraph to the Model definition section (lines 128–136) :

"In the model, we formally assume that all cells are identical and all variability follows from stochastic transitions between cell states. In recent years, it has been demonstrated that population heterogeneity is mainly responsible for observed variability in signal transduction [11–13]. However, in our case (as in many other cases) the two types of noise (intrinsic and extrinsic) have similar consequences. Stochasticity in front propagation is a consequence of variability in state-to-state transition times; this variability may have both intrinsic and extrinsic origins. The key difference is that under intrinsic noise, the front is expected to be perturbed in random locations, while under extrinsic noise, some specific locations in the monolayer will more likely be the source of front perturbation."

Reviewer #3

1 Synopsis

The authors study a cellular automaton model representing the propagation of spatiotemporal waves of MAPK/ERK activity in a monolayer of interacting cells. The model resembles a stochastic SIR (susceptible, infected, recovered) epidemic model (with loss of immunity) on a lattice [1], or the classic Bak-Chen-Tang (BCT) forest re model [2], albeit with additional substates. The authors numerically study the propagation, branching, and failure of wavefronts seeded at one end of a long, narrow two-dimensional domain, with a particular focus on the effects of changing the domain width. In order to study how well signals propagate in the simulated system, the authors experiment with train of pulses initiated either by a rhythmic pattern of excitation at one end of the domain, or else by a random binary sequence of excitations with different time intervals. In the latter case they estimate an information transmission rate, roughly the mutual information between pulse initiation at one end of the domain and pulse arrival at the other end of the domain.

On the one hand, the system studied by the authors is interesting as a stochastic cellular automaton system, and the authors conduct a very thorough study, although some of the details of their analysis could be presented more clearly. On the other hand, it is not very clear why the noisy propagation of a traveling wave through a long thin strip of tissue is relevant to understanding the functioning of a MAPK/ERK signaling biologically; moreover, the rationale for using an information theoretic framework does not seem very well motivated.

Since my expertise is more in modeling and applications of information theory in neural systems than in MAPK/ERK systems, I will mainly focus this report on the information theoretic aspects of the system.

2 Major issues

1. It is not clear why estimating information rates is relevant to the problem of understanding MAPK/ERK signaling in the kind of system the authors consider. According to the paper, MAPK/ERK activity is important for “long-range communication” in tissues regenerating after, say, injury. (The authors estimate a maximal bitrate for their model system at around 0.5 bit/hour, cf. Fig. 4E. Is there any biological significance to this number?) The presence of spatiotemporal traveling waves does not by itself imply that there is a “communications system” at work[1]. 1 Is there really a “receiver” in the MAPK/ERK system that is waiting to receive a signal transmitted through the tissue from a spatially distant “sender” selecting a message from a stochastic ensemble of possible messages? Or is the MAPK/ERK system merely another of many examples of a spatially extended excitable system that can support traveling waves? In order to justify the use of information rates as a quantitative metric of the behavior of the MAPK/ERK system, the authors should better motivate why the information theoretic framework is relevant to this system[2]. If the important function is propagation of MAPK/ERK activation more than

> *communication of a "message," then another framework such as signal-detection theory [6] or ideal-observer analysis [7] might be more relevant.*
>
> *[1] For example: There are propagating waves of hypoxia and/or ionic imbalance in models of spreading depression and epileptic activity [3, 4]. The brain, as an organ, is involved in information and communication. But I am not aware of anyone trying to estimate the rate at which a propagating spreading depression wavefront conveys information from one part of (say) the hippocampus to another. Why not? Because conveying information is not the purpose of spreading depression. (According to [3], SD helps prevent seizures, so one might consider that to be its purpose.)*
>
> *[2] Cf. Shannon's classic polemic [5].*

Response: As discussed in the Introduction, ERK activity waves "coordinate collective migration of epithelial cells [1,2]. Excitable ERK activity waves have been shown to control the rate of scale regeneration in zebrafish [3]". Indeed, ERK activity waves propagate outward from the wound edge and trigger both cell proliferation and migration toward the wound, which is essential for healing. These waves are required to propagate in a coordinated manner to direct the migrating cells.

Nevertheless, we are not able to bridge the estimated information transmission rate with the observed behavior. Rather, we think that the estimated value, ~0.5 bit/hour, imposes an upper limit on the complexity and coherency of distant cells' behavior.

It is hardly an explanation for your concerns (which in part we share), but a number of researchers employ information theory to determine the amount of information that can be transmitted by cellular systems (Cheong *et al*., 2011, DOI: 10.1126/science.1204553; Selimkhanov *et al*., 2014, DOI: 10.1126/science.1254933) or the rate of information transmission (Mattingly *et al*., 2021, DOI: 10.1038/s41567-021-01380-3), and our motivation was to extend these results to cell populations.

> ***2.** The calculation of "information rates" for long thin strips of tissue seems like a dubious form of quantification because (if I understand correctly) in real systems (which are not thin strips but more likely to be tissue regions that are both broad and long?) the propagating wave fronts break up into chaotic spiral front patterns that are not "carrying information" from a sender to a receiver. Do the authors have specific systems in mind where the "tissue channel" connecting the tissue where activation waves are initiated and propagate linearly to another region where they are somehow needed to arrive in a regular temporal sequence? In short, is there a biological system that looks and acts like the authors' model system (a long thin strip)? If not, the biological relevance of the model and its analysis might not be sufficient for this journal.*

Response: Yes, the study is biased towards narrow channels, because we found that in these channels the information transmission rate is highest, which in our opinion is a point of interest and may give new biological insights. Narrow structures such as capillaries and lymphatic vessels exist, and recently pulsatile ERK signaling was observed in regenerative angiogenesis (Okuda et al., DOI: 10.7554/eLife.62196). Nevertheless, in response to your concerns, we added a new subsection *Front propagation in broad channels* (lines 505-539) to discuss the parameter regime in which unperturbed front propagation in broad channels is possible as well. In the case of broad channels, the information rate is not an adequate measure as front spawning may lead to a long-lasting chaotic phase. Therefore, we ask how

many pulses can be propagated before the first spawning event and show that the expected number of fronts increases exponentially with refractory time. This implies that a relatively modest change in parameters allows for efficient front propagation also in broad channels. Consequently, the behavior of the system may be qualitatively different in various conditions (cell type, density, temperature, etc.).

> ***3.*** *A single-use communications channel is specified by a source random variable X with distribution $P_X(X)$, a receiver random variable Y and a conditional probability distribution P(Y | X) that characterizes the channel. For a given choice of the input distribution P_X one obtains the mutual information I(X : Y) = H(Y) − H(Y | X) = H(X) − H(X | Y). The capacity of the channel is the maximum (or, technically, the supremum) of I(X : Y) over all allowable input distributions P_X. In a repeated-use communications channel, like the system considered by the authors, the input is a stochastic process X_t, one considers the mutual information rate $\lim_{T\to\infty} 1/T\, I(X^T_0 : Y^T_0)$ where X^T_0 is the trajectory of the process X_t over the time interval $0 \le t \le T$. The capacity is the largest value of the mutual information rate obtained over all allowed input processes (e.g. subject to some constraint). For a system like that considered by the authors one of the difficulties in studying either the information rate or the capacity is intersymbol interference, in which transmission of one symbol can be corrupted by the transmission of a later or earlier symbol. In studying the information rate of their channel, the authors restrict the input to a binary sequence of activations with probability p_{act} = 1/2 at fixed time steps. But this is only one of many possible input processes the authors could consider. The problem is that limiting to a single input process, one does not know if the information rate is anywhere near the best you can obtain for the channel.*
>
> *It is true that the authors vary the time interval T_{slot} between symbol transmissions (the kth symbol being either an activating pulse or the absence of an activating pulse at time $T_k = t_0 + kT_{slot}$), and estimate the MI rate as a function of T_{slot}. However, I think it would be important for the authors to consider a broader class of inputs. I offer two suggestions, the first perhaps more practical than the second.*
>
> *(a) Vary p_{act}. Particularly because of intersymbol interference, for a fixed value of T_{slot}, sending activation pulses with probability p_{act} < 1/2 would reduce the entropy rate of the source X_t but it would also reduce the intersymbol interference. These two competing effects could lead to the optimal pact being considerably less than 1/2.*
>
> *(b) Since the authors are operating their channel in continuous time, an even more general (but quite natural) class of input processes would be Poisson processes with a specified rate λ_{act}. In this case, instead of making the artificial assumption that the sender and receiver have access to a shared noiseless clock, the timing of activations at the "sending" end of the channel would be a random increasing sequence of times $0 < T_1 < T_2 < . . ..$*

Response: Indeed, in this paper, we did not perform protocol optimization (apart from adjusting the time interval T_{slot}) and, therefore, our estimates provide only a lower bound of the channel capacity. The motivation for this approach was to keep the methodology as simple as possible so that it does not cover the analysis of stochastic effects that limit information transmitted by excitable waves.

In the revised version, we follow your suggestion and add S1 Text with S8 Fig (referenced in lines 413-421), in which we consider binary protocols with p_{act} (or q in our notation) not confined to ½. Indeed, in the case of short channels (L = 30), such a protocol can be optimized (by adjusting simultaneously q and T_{slot}) to transmit information at about 20% higher rate than the protocol with q = ½.

Based on our previous study on information transmitted in the MAPK/ERK system in single cells (Nałęcz-Jawecki *et al*., 2023, DOI: 10.1371/journal.pcbi.1011155), we may expect that for very short channels (in which the transit time variance $\sigma^2_{transit}$ is small) the optimal protocol would follow the Poisson process but with intervals shorter than the refractory time excluded. This exclusion is beneficial, as fronts sent at short intervals are very unlikely to get transmitted (and thus carry little information), but may mess up in the channel. Nevertheless, setting slot interval T_{slot} below $\sigma_{transit}$ comes with no further increase in bitrate, and therefore continuous-time protocols (that can be modeled with $T_{slot} \to 0$, keeping q/T_{slot} = const) would not significantly outperform the protocols with $T_{slot} \approx \sigma_{transit}$.

> ***4.** Given the restriction to binary input sequences at clocked intervals, the procedure used by the authors to estimate the information transmission rate seems to require further clarification and justification.*
>
> *(a) The mean front velocity is not well defined (paragraph preceding eq. (7)). I think the authors actually mean the conditional mean front velocity, i.e. conditioned on the front arriving at the far end of the channel. (If a front stops propagating in mid-channel, is its velocity zero? Is it included in calculating the mean front velocity?) Similarly,* $\langle \tau_{transit} \rangle$ *should be better defined. Since there is a chance of a front never arriving, the expected transit time is probably infinite. That is, for any number a > 0, one has the inequality* $E[\tau_{transit}] \geq E[\tau_{transit} \mid \tau_{transit} < a]\, P(\tau_{transit} < a) + a(1 - P(\tau_{transit} < a))$. *If there is a finite chance that a front never arrives, then* $P(\tau_{transit} < a) < 1$ *for all a. In this case, taking the limit as* $a \to \infty$ *shows that the expected transit time must be* $+\infty$. *The remedy is to consider the conditional transit time, that is, the mean transit time conditioned on the event that the pulse arrives within a finite time:*
>
> $$\langle \tau_{transit} \rangle \equiv E[\tau_{transit} \mid \tau_{transit} < \infty].$$
>
> *If the transit time has a density* f_{trans} *then* $\langle \tau_{transit} \rangle = \int_0^\infty t f_{trans}(t)\, dt \,/ \int_0^\infty f_{trans}(t)\, dt$ *where the denominator is* $P(\tau_{transit} < \infty) < 1$.

Response: Yes, we use transit time conditioned on the front reaching end of the channel. We added a note on this to the *Methods → Numerical data analysis → Front speed and transit time variance* section (lines 662–664).

> *(b) The description of the procedure:*
> *"...for each ith slot, the difference* Δt_i *between the closest arrival time (to the expected arrival time of the ith transmitted symbol) and the expected arrival time was measured" is problematic. Consider the following scenario: slot k − 1 sends "0"; slot k sends "1"; slot k + 1 sends "1". Suppose both the kth and k + 1st slots' wavefronts fail to propagate, but a disruptive event near the time of the k − 1st slot leads to an ectopic front arrival at a time*

somewhere between the expected arrival times for the kth and k + 1st slots. Then according to the procedure, the ectopic arrival would be counted as an arrival originating from both the kth and the k + 1st slots. It would contribute twice to the formula (7). Further clarification, or possibly a correction, is required.

Response: During our procedure, we do not explicitly associate observed arrivals with sent fronts. In fact, the whole procedure described in this section is a way to compute the mutual entropy between two random variables: the symbols $S \in \{0,1\}$ determining whether a front was initiated in a given slot (at time t) and the temporal distance Δt from the expected arrival time ($t + L/v$, where L is the channel length and v the average front velocity) to the nearest observed arrival. The expected arrival time is associated with each slot, i.e. also in the case when no pulse was initiated in the slot. This might have been unclear, therefore we rewrote this section of Methods (lines 697-727) and modified the way how we refer to it in the main text (lines 311-327). We also stressed that this approach is different from how we estimate the information loss in the semi-analytical estimates (lines 768-771). In S5 Fig we show histograms of Δt for S=1 and S=0; one can see that for T_{slot} =150 min these histograms are easily separable, while for T_{slot} = 90 min there is a significant overlap. This implies a higher MI_{slot} for T_{slot} =150 min.

(c) After conducting an ensemble of simulations, then for each time slot the authors count the occurrences of certain time differences Δt between each of a list of expected arrival times and the nearest actual front arrival time. If a time difference occurs a certain number of times (k = 25 before eq. (7)) or more, then a conditional entropy $H(S_i \mid \Delta t)$ associated with the ith slot is calculated according to one formula (7) but if the time difference occurs fewer than k times, then the authors use a different formula for $H(S_i \mid \Delta t)$. And on the next page we read "In cases where the MIslot estimation was based on the closest front and its predecessor k = 15 was used as it provides better performance." The authors do not sufficiently explain the use of two different formulas for the conditional entropy, one above k and another below k. Why not use one of the formulas throughout, for consistency? They do not explain sufficiently why k was chosen to be 25. What happens if k is 10, or 50, or some other arbitrary number? They do not adequately explain why they change k from 25 to 10 in some situations. Too many elements of this procedure seem to be ad hoc*.*

Response: As mentioned above, the whole procedure associated with formula (7) is simply a way to compute the mutual information between S and Δt. We achieve that by estimating the conditional distribution S given Δt. In cases when a given difference Δt (binned with one-minute resolution) occurs sufficiently many times (at least k) in the dataset, we are able to compute $p_1 = P(S = 1 \mid \Delta t)$ naively by counting the occurrences $n_s = \#\{(s_i, \Delta t_i): s_i = s, \Delta t_i = \Delta t\}$ for s = 0,1 and taking the ratio $n_1/(n_0 + n_1)$. However, if there are not enough occurrences of a given Δt in the dataset, such estimation would be inaccurate and therefore we "interpolate" $P(S = 1 \mid \Delta t)$ by counting not only the cases where Δt_i is exactly equal Δt, but also other cases with Δt_i as close as possible to Δt so that the total number of cases is at least k. We chose $k = 25$ for 1-dimensional Δt and $k = 15$ for multidimensional Δt as for these thresholds the MI estimate was the highest. However, we agree with your comment that consistency is important and now set k = 25 in all cases (this slightly lowered the estimates in S9 Fig in S2 Text in the revised manuscript). We also provided an additional explanation in the Methods section (lines 716–718).

3 Minor issues

It would have been convenient if the authors had numbered the pages and lines of their manuscript.

Response: Thank you for the remark and apologies for the inconvenience, this was our neglect. We added line numbering, as requested.

1. Author summary: "However, random effects on THE single-cell level..."

Response: Corrected (line 25).

2. Author summary: "the refractory time ... must be long with respect TO THE TIME needed for cell activation."

Response: Corrected (line 31).

3. Introduction: "This is the case of spatiotemporal" → "This is the case FOR spatiotemporal"

Response: Corrected (line 37).

4. Results / Model definition: Please state early in the modeling section whether the implementation of the model is in continuous time or discrete time. Until the mention of Gillespie's algorithm later, in the Methods section, it was ambiguous.

Response: The model is in continuous time; we added this information at the beginning of the section (line 82).

5. Results / Model definition: From the author's presentation it appears they assume that the rate of spontaneous excitation $Q \rightarrow E_1$ is identically zero in the absence of stimulation from neighboring inducing cells. Is that realistic? In some communications channels (e.g. Kabanov's Poisson channel [10], which is a more elementary point-process channel, like the one the authors consider) the capacity is infinite unless there is a dark current, i.e. a finite false positive rate, or finite chance of receiving a signal even if one was not sent. Please clarify the assumption that there is not spontaneous excitation.

Response: Spontaneous ERK activation is indeed observed *in vitro* (Albeck *et al*., 2013, DOI: 10.1016/j.molcel.2012.11.002; Aoki *et al*., 2013, DOI: 10.1016/j.molcel.2013.09.015), and the frequency of such events varies considerably depending on specific conditions like cell line, culture medium, etc. We decided to exclude it from the model for simplicity, and due to the fact that in reality, spontaneous activation of a single cell or small group of cells is not likely to induce a wavefront that would propagate for more than a few cell layers (see

Gagliardi *et al*., 2021, DOI: 10.1016/j.devcel.2021.05.007), probably due to dissipation of the mechanical tension (which is required for wave propagation).

Note that the spontaneously initiated fronts act somehow similarly to forward-spawned fronts, which are present in our model. Nevertheless, even in the absence of such events, the channel capacity would be finite, as the temporal resolution is limited by the transit time distribution.

> ***6.*** *Results / Propagation of a single front / Propagation failure and front spawning: Below eq. (1), "On the contrary" should be "In contrast".*

Response: This has been corrected (line 213).

> ***7.*** *Results / Optimal channel width: Eq. (3) is not adequately explained. On the one hand, it seems clear that the probability that a front fails to make it through the channel will depend on the rates of the two failure modes (front stops propagating spontaneously; front is annihilated by a backpropagating front). So the probability of one front in a train failing to reach the other side may be a function of both λ_{fail} and λ_{spawn}. But why should it be simply the sum of these two rates? Is there an unstated assumption that the two failure modes are mutually exclusive? Please justify this expression more carefully.*

Response: Formula (3) is an approximation – we neglected the possibility that more than one front is spawned in a single event and that a backward front is subjected to another disruptive event before colliding with the subsequent front. The approximate formula is sufficient to determine the optimal channel width, because λ_{fail} *decreases very fast* with the channel width. We alleviate the first assumption in section *Semi-analytical predictions based on phenomenological analysis*, when the expected number of back spawned fronts in a single spawning event is taken into account. Additional explanation was added in lines 225 - 229.

> ***8.*** *Results / Optimal channel width: Does "uninterrupted front propagation" refer to the propagation of a single isolated front, a single front in an infinite chain, or an entire infinite chain of fronts? Each of these events has different probabilities. In particular, the probability of "uninterrupted front propagation" is strictly zero, if it refers to an infinite series of fronts. At least one of a long series is sure to fail. Please clarify the meaning of the phrase.*

Response: We meant uninterrupted passing of a front in a front series. An explanation has been added to the manuscript (line 232).

> ***9.*** *Results / Propagation of periodically initiated fronts: "fronts trains" should be "front trains" at the end of this paragraph.*

Response: Corrected (line 309).

> ***10.*** *Results / Information transmission rate / Numerical results: “To assess the bitrate” should be “To estimate the bitrate”.*

Response: Corrected (line 312).

> ***11.*** *Methods / Information transmission rate – numerical estimation: “To measure the information transmission rate” should be “To estimate the information transmission rate.”*

Response: Corrected (line 698).

> ***12.*** *Methods / Information transmission rate – semi-analytical predictions: Is eq. (9) the probability of a single, isolated front being eliminated? Or the probability of any front in a long train being eliminated? Or of a single front in a long train?*

Response: A single front in a long train. Corrected (line 729).

> ***13.*** *Methods / Information transmission rate – semi-analytical predictions: In the confusion matrix, eq. (10), setting the top right entry to zero means that there is no possibility of spontaneous activations occurring. This means that the activation rate is strictly zero unless there is a neighboring cell in the inducing state. Is this biologically accurate? (Cf. question 5 above.)*

Response: See the answer to minor issue ***5.***

> ***14.*** *Methods / Information transmission rate – semi-analytical predictions: The authors have two expressions for MI_{slot}, both given by eq. (11) but with two different confusion matrices (10) and (17). In order to see the effects of replacing matrix (10) with matrix (17), did the authors compare the two different values of MI_{slot}?*

Response: Actually, we do compare the two formulas in Figs 4G–I: dotted line is computed with matrix (11) [previously (10)] with p taken from Eq (16) [previously (15)], while the dashed uses matrix (18) [previously (17)].

[Reviewer's] References

[1] David R De Souza and Tânia Tome. Stochastic lattice gas model describing the dynamics of the sirs epidemic process. Physica A: Statistical Mechanics and its Applications*, 389(5):1142–1150, 2010.*

[2] Per Bak, Kan Chen, and Maya Paczuski. Solitons in the one-dimensional forest fire model. Physical Review Letters*, 86(11):2475, 2001.*

[3] Yina Wei, Ghanim Ullah, and Steven J Schiff. Unification of neuronal spikes, seizures, and spreading depression. Journal of Neuroscience*, 34(35):11733–11743, 2014.*

[4] Bas-Jan Zandt, Bennie ten Haken, Michel JAM van Putten, and Markus A Dahlem. How does spreading depression spread? physiology and modeling. Reviews in the Neurosciences*, 26(2):183–198, 2015.*

[5] Claude E Shannon. The bandwagon. IRE transactions on Information Theory*, 2(1):3, 1956.*

[6] David Marvin Green, John A Swets, et al*.* Signal detection theory and psychophysics*, volume 1. Wiley New York, 1966.*

[7] Eric E Thomson and William B Kristan. Quantifying stimulus discriminability: a comparison of information theory and ideal observer analysis. Neural computation*, 17(4):741–778, 2005.*

[8] Peter J Thomas and Andrew W Eckford. Capacity of a simple intercellular signal transduction channel. IEEE Transactions on information Theory*, 62(12):7358–7382, 2016.*

[9] JA McFadden. The entropy of a point process. Journal of the society for industrial and applied mathematics*, 13(4):988–994, 1965.*

[10] Yu M Kabanov. The capacity of a channel of the poisson type. Theory of Probability & Its Applications*, 23(1):143–147, 1978.*
