# Supplementary material for: Information transmission in a cell monolayer: A numerical study
Source: PLoS Comput Biol. 2025 Feb 21;21(2):e1012846. doi: 10.1371/journal.pcbi.1012846 (PMC11902151; doi:10.1371/journal.pcbi.1012846)
Supplement: Attachment — Submitted filename: report.pdf [file pcbi.1012846.s008.pdf]

Report on “Information transmission in a cell monolayer: A numerical study” by Nalecz-Jawecki et al.

January 7, 2025

1 Summary

In my first report on “Information transmission in a cell monolayer: A numerical study,” I raised four main concerns:

1. The biological significance of estimating information rates for the MAPK/ERK system is not clear.

2. Even if information transmission was a relevant aspect of this system, estimating such rates in long thin strips lacks relevance since signal propagation occurs throughout the tissue, not in narrow tissue channels.

3. The authors did not adequately account for inter-symbol interference (successive bits in a sequence overwriting each other) although they show this is likely to occur some of the time and really only get a rough estimate of the capacity of their “channel”.

4. The ad hoc procedure the authors use for estimating information arrival seems unjustified (e.g. there is an arbtirary parameter “k” set to different values under different circumstances without sufficient explanation why.)

The authors have completed substantial work in response to all three referees’ concerns that have clearly strengthened the paper. For example, I am intrigued to see that by varying the binary sending probability (q) they have demonstrated an increase in the information rate relative to the $q = 1/2$ rate. In particular, the authors have adequately addressed my technical concerns raised in points 2-4 above. My remaining reservations are not technical so much as philosophical. Perhaps there is no good answer to my main concern, “why is information theory relevant to this system?” So I will leave it to the editor’s judgment whether this conceptual gap – which the authors acknowledge in their reply (but not in the revised paper itself?) – is important enough to prevent publication. If one grants that information metrics are worth pursuing for their own sake (which I do not, but I recognize

I may speak for an intellectual minority) then I think the paper is fit for publication in its revised form.

Let me now go point-by-point.

2 Major points

1. I claimed that the biological significance of information rates for the MAPK/ERK system is not clear.

Note: The authors refer to the well known paper by Cheong et al (2011), and others, as a precedent. In the Cheong paper the pathway they studied led in a fairly clear way to a yes/no decision on the part of the "receiver", and the information channel analogy made a lot of sense to me. In Mattingly et al (2021) the authors relate information processing bounds to accuracy in performance of a specific behavior (accuracy of gradient climbing by chemotaxing bacteria). I do not dispute the importance of the ERK system for wound healing, but if what the interacting cells accomplish is "coordination," then it would make more sense to introduce a coordination metric rather than an information metric per se. In biology, not all "information" is equally useful to the organism.[1] The authors' previous publication in this journal showed that the localized MAPK/ERK system had a bit rate of not less than 6 bits per hour, while the present system seems to be able to reach around 6 bits per ten hours. What do these numbers mean biologically? If the local MAPK/ERK system could only"transmit" 5 bits per hour instead of 6 bph, would the zebra fish not be able to recover from small injuries? Would its fins decay and its tail fall off?

And yet, as I mentioned above, this is a philosophical rather than a technical concern.

2. Even if information transmission was a relevant aspect of this system, I was concerned that estimating such rates in long thin strips lacked relevance since signal propagation occurs throughout the tissue, not in narrow tissue channels.

Note: The authors have addressed this point as best they could, by examining wider channels, and by pointing out that there *are* some tubular structures (e.g. blood vessels) for which their geometry would be relevant.

3. I was concerned that the authors did not adequately account for inter-symbol interference (successive bits in a sequence overwriting each other) although they show this is likely to occur some of the time and really only get a rough estimate of the capacity of their "channel".

Note: The authors have adequately addressed these concerns.

4. The ad hoc procedure the authors use for estimating information arrival seems unjustified (e.g. there is an arbtirary parameter "k" set to different values under different circumstances without sufficient explanation why.)

[1] For a study that indirectly suggests how to quantify "useful" vs "useless" information, see Moffett, Alexander S., and Andrew W. Eckford. "Minimal informational requirements for fitness." Physical Review E 105.1 (2022): 014403.

Note: The authors have adequately clarified their procedures.

3 Minor points

1. Circa lines 508-509 the authors write "In broad channels of $W \gtrsim 15$, front spawning events frequently seed multiple fronts (Fig 2DE) giving rise to chaotic front propagation patterns (as shown in S2 Fig and S4 Video), which can block information transmission for very long times. Thus, *for such channels information transmission rate is not well defined,...*"

   On the contrary, in this scenario the information transmission rate IS well defined – and it is either identically zero (a finite number of bits b can be sent in an infinite amount of time, so the rate is $\lim_{T\to\infty)}(b/T) = 0$) or else it is very close to zero. Note the conclusion of the sentence "and one should rather ask about the number of fronts that can be transmitted before chaotic patterns develop due to spawning event(s)." is fine as it is, but the claim that the information rate is "undefined" should be corrected.

2. On lines 704-705, the authors write $t_{\text{expected}} = t_{\text{slot}} + \langle \tau_{\text{transit}} \rangle = t_{\text{slot}} + L/\langle v \rangle$. However it is *not* in general true that $\langle \tau_{\text{transit}} \rangle = L/\langle v \rangle$. The reciprocal of the average is not (usually) the average of the reciprocal. Put another way, suppose f is the density of a nonnegative random variable (here, τ_{transit}) and suppose we define a new random variable $V = L/\tau_{\text{transit}}$. Then the identity implicitly asserted on lines 704-705,

   $$\int_0^\infty f(t)t\,dt = \frac{1}{\int_{t=0}^\infty f(t)\frac{1}{t}\,dt}$$

   is generally false. I am willing to believe that the error resulting from this approximation is small, but I wish the authors would be more precise in their notation.
