# Supplementary material for: Information transmission in a cell monolayer: A numerical study
Source: PLoS Comput Biol. 2025 Feb 21;21(2):e1012846. doi: 10.1371/journal.pcbi.1012846 (PMC11902151; doi:10.1371/journal.pcbi.1012846)
Supplement: Attachment — Submitted filename: Nalecz-Jawecki_et_al_Response_to_Reviewers_auresp_2.pdf [file pcbi.1012846.s009.pdf]

Response to Reviewers

Reviewer #3

1. The authors refer to the well-known paper by Cheong et al (2011), and others, as a precedent. In the Cheong paper the pathway they studied led in a fairly clear way to a yes/no decision on the part of the "receiver", and the information channel analogy made a lot of sense to me. In Mattingly et al (2021) the authors relate information processing bounds to accuracy in performance of a specific behavior (accuracy of gradient climbing by chemotaxing bacteria). I do not dispute the importance of the ERK system for wound healing, but if what the interacting cells accomplish is "coordination", then it would make more sense to introduce a coordination metric rather than an information metric per se. In biology, not all "information" is equally useful to the organism. The authors' previous publication in this journal showed that the localized MAPK/ERK system had a bit rate of not less than 6 bits per hour, while the present system seems to be able to reach around 6 bits per ten hours. What do these numbers mean biologically? If the local MAPK/ERK system could only "transmit" 5 bits per hour instead of 6 bph, would the zebrafish not be able to recover from small injuries? Would its ns decay and its tail fall off?
   And yet, as I mentioned above, this is a philosophical rather than a technical concern.

Response: As suggested by the Editor, we added a new, currently penultimate, paragraph to Discussion, discussing the above concerns of Reviewer. We think that, in our case, the information metric is more adequate than the coordination metric. As we write in the last revised paragraph of Discussion, "bitrate limits temporal resolution, complexity of a desired behavior, and the extent of cell coordination". In our case, ERK waves are better coordinated (and thus less perturbed) when sent at longer intervals, but longer intervals imply less frequent ERK activation and correspondingly slower cell proliferation and movement. Maximization of the bitrate reflects the tradeoff between cell coordination and wave frequency and, therefore, is a better measure than coordination alone.

Minor comments

1. Circa lines 508-509 the authors write "In broad channels of $W \gtrsim 15$, front spawning events frequently seed multiple fronts (Fig 2DE) giving rise to chaotic front propagation patterns (as shown in S2 Fig and S4 Video), which can block information transmission for very long times. Thus, *for such channels information transmission rate is not well defined*,…"
   On the contrary, in this scenario the information transmission rate IS well defined and it is either identically zero (a finite number of bits *b* can be sent in an infinite amount of time, so the rate is $\lim_{T\to\infty} \frac{b}{T} = 0$) or else it is very close to zero. Note the conclusion of the sentence "and one should rather ask about the number of fronts that can be transmitted before chaotic patterns develop due to spawning event(s)" is fine as it is, but the claim that the information rate is "undefined" should be corrected.

Response: Indeed, this is a valid remark. We replaced "is not well defined" with "is close to zero" in line 509.

2. On lines 704-705, the authors write $t_{expected} = t_{slot} + \langle\tau_{transit}\rangle = t_{slot} + L/\langle v\rangle$. However it is *not* in general true that $\langle\tau_{transit}\rangle = L/\langle v\rangle$. The reciprocal of the average is not (usually) the average of the reciprocal. Put another way, suppose *f* is the density of a nonnegative random variable (here, transit) and suppose we de ne a new random variable $V = L/\tau_{tranist}$. Then the identity implicitly asserted on lines 704-705,

$$\int_{-}^{\infty} f(t)\, t\, dt = \frac{1}{\int_{-}^{\infty} f(t)\frac{1}{t}\, dt}$$

is generally false. I am willing to believe that the error resulting from this approximation is small, but I wish the authors would be more precise in their notation.

Response: The Reviewer is right in saying that $\langle\tau_{transit}\rangle$ is not equal $L/\langle v\rangle$ as, in general, the reciprocal of the average does not need to be equal to the average of the reciprocals. We corrected the mentioned formula by changing $L/\langle v\rangle$ to $L\langle v^{-1}\rangle$ and adjusted the text and S1 Fig accordingly. As we previously used the harmonic mean to compute average velocity $\langle v\rangle$, the numerical value of $t_{expected}$ computed according to the formula $t_{expected} = t_{slot} + \langle\tau_{transit}\rangle = t_{slot} + L/\langle v\rangle$ (which is now correctly written as $t_{expected} = t_{slot} + \langle\tau_{transit}\rangle = t_{slot} + L\langle v^{-1}\rangle$) is correct.
